# Supplementary material for: Carbon Nanotube Films with Fewer Impurities and Higher Conductivity from Aqueously Mono-Dispersed Solution via Two-Step Filtration for Electric Heating
Source: Nanomaterials (Basel). 2024 May 22;14(11):911. doi: 10.3390/nano14110911 (PMC11173405; doi:10.3390/nano14110911)
Supplement: Supplementary file 1 [file nanomaterials-14-00911-s001.zip › nanomaterials-2993419-supplementary.pdf]

Carbon Nanotube Films with Fewer Impurities and Higher Conductivity from Aqueously Mono-Dispersed Solution via Two-Step Filtration for Electric Heating

Yingying Chu [1], Ling Sun [1, 2*], Jing Wang [1], Zhaoyang Han [1], Chenyu Wei [1], Changbao Han [1], Hui Yan [1]

1. Key Laboratory of Advanced Functional Materials, Ministry of Education, Faculty of Materials and Manufacturing, Beijing University of Technology, Beijing 100124, China

2. Beijing Guyue New Materials Research Institute, Beijing University of Technology, Beijing 100124, China

*Corresponding author. E-mail: sunling@bjut.edu.cn (L. Sun)

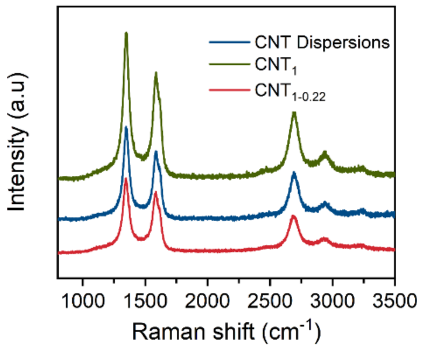

Figure S1 The Raman Spectrometer of CNTs dispersions, CNT_1 film, and $CNT_{1-0.22}$ film.

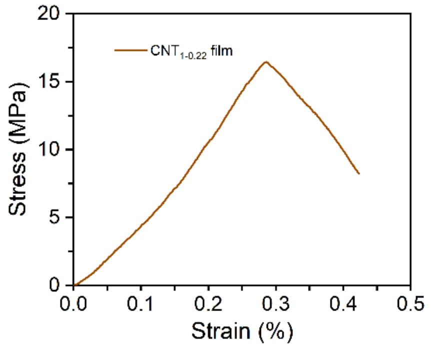

Figure S2 Stress-strain curves of $CNT_{1-0.22}$ film.

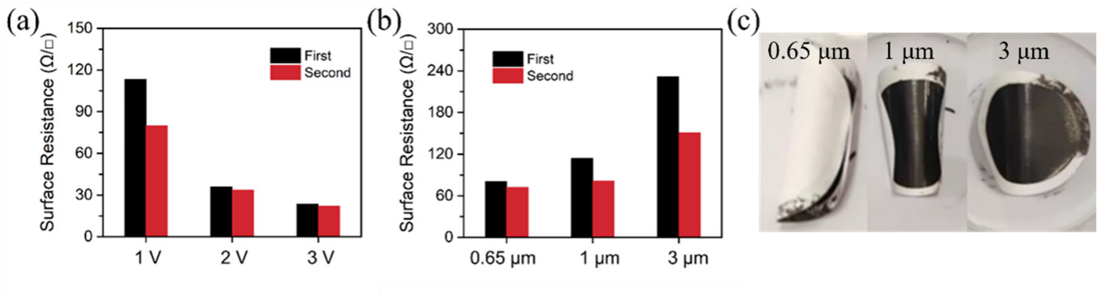

Fig.ure S3 (a) The surface resistance of different volumes of CNTs dispersions; (b) The surface resistance of 100 ml CNTs dispersions filtered through different pore size membranes; (c) Photographs of 300 ml of CNTs dispersions filtered through membranes of different pore sizes.

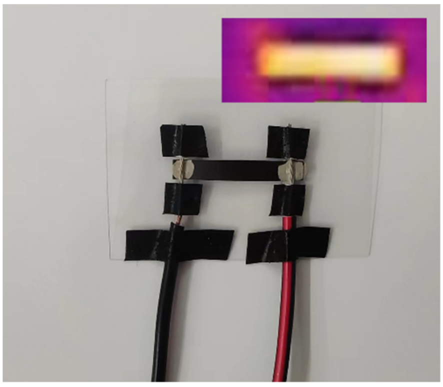

Figure S4 A schematic diagram of the assembled CNTs heating film and the infrared image of heat generation.

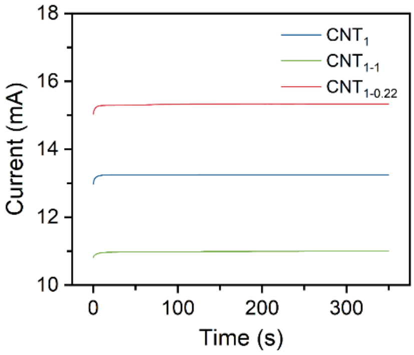

Figure S5 Current changes at applied voltages of 10 V for the CNT_1 film, $CNT_{1\text{-}1}$ film and $CNT_{1\text{-}0.22}$ film.

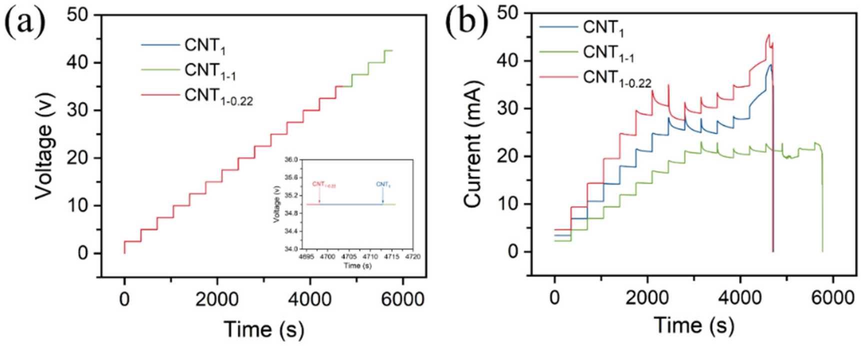

Figure S6 Heating performance of CNTs films under increasing voltage: (a) Curve voltage over time; (b) Curve current over time.
